# Application of fed-batch strategy to fully eliminate the negative effect of lignocellulose-derived inhibitors in ABE fermentation

**DOI:** 10.1186/s13068-024-02520-6

**Published:** 2024-06-25

**Authors:** Barbora Branska, Kamila Koppova, Marketa Husakova, Petra Patakova

**Affiliations:** Department of Biotechnology, University of Chemistry and Technology Prague, Technická 5, 16628 Prague, Czech Republic

**Keywords:** ABE fermentation, Butanol, Clostridium, Lignocellulose hydrolysate, Fed batch, Wheat straw, Inhibitors, Ferulic and coumaric acid, Salinity

## Abstract

**Background:**

Inhibitors that are released from lignocellulose biomass during its treatment represent one of the major bottlenecks hindering its massive utilization in the biotechnological production of chemicals. This study demonstrates that negative effect of inhibitors can be mitigated by proper feeding strategy. Both, crude undetoxified lignocellulose hydrolysate and complex medium supplemented with corresponding inhibitors were tested in acetone–butanol–ethanol (ABE) fermentation using *Clostridium beijerinckii* NRRL B-598 as the producer strain.

**Results:**

First, it was found that the sensitivity of *C. beijerinckii* to inhibitors varied with different growth stages, being the most significant during the early acidogenic phase and less pronounced during late acidogenesis and early solventogenesis. Thus, a fed-batch regime with three feeding schemes was tested for toxic hydrolysate (no growth in batch mode was observed). The best results were obtained when the feeding of an otherwise toxic hydrolysate was initiated close to the metabolic switch, resulting in stable and high ABE production. Complete utilization of glucose, and up to 88% of xylose, were obtained. The most abundant inhibitors present in the alkaline wheat straw hydrolysate were ferulic and coumaric acids; both phenolic acids were efficiently detoxified by the intrinsic metabolic activity of clostridia during the early stages of cultivation as well as during the feeding period, thus preventing their accumulation. Finally, the best feeding strategy was verified using a TYA culture medium supplemented with both inhibitors, resulting in 500% increase in butanol titer over control batch cultivation in which inhibitors were added prior to inoculation.

**Conclusion:**

Properly timed sequential feeding effectively prevented acid-crash and enabled utilization of otherwise toxic substrate. This study unequivocally demonstrates that an appropriate biotechnological process control strategy can fully eliminate the negative effects of lignocellulose-derived inhibitors.

**Graphical Abstract:**

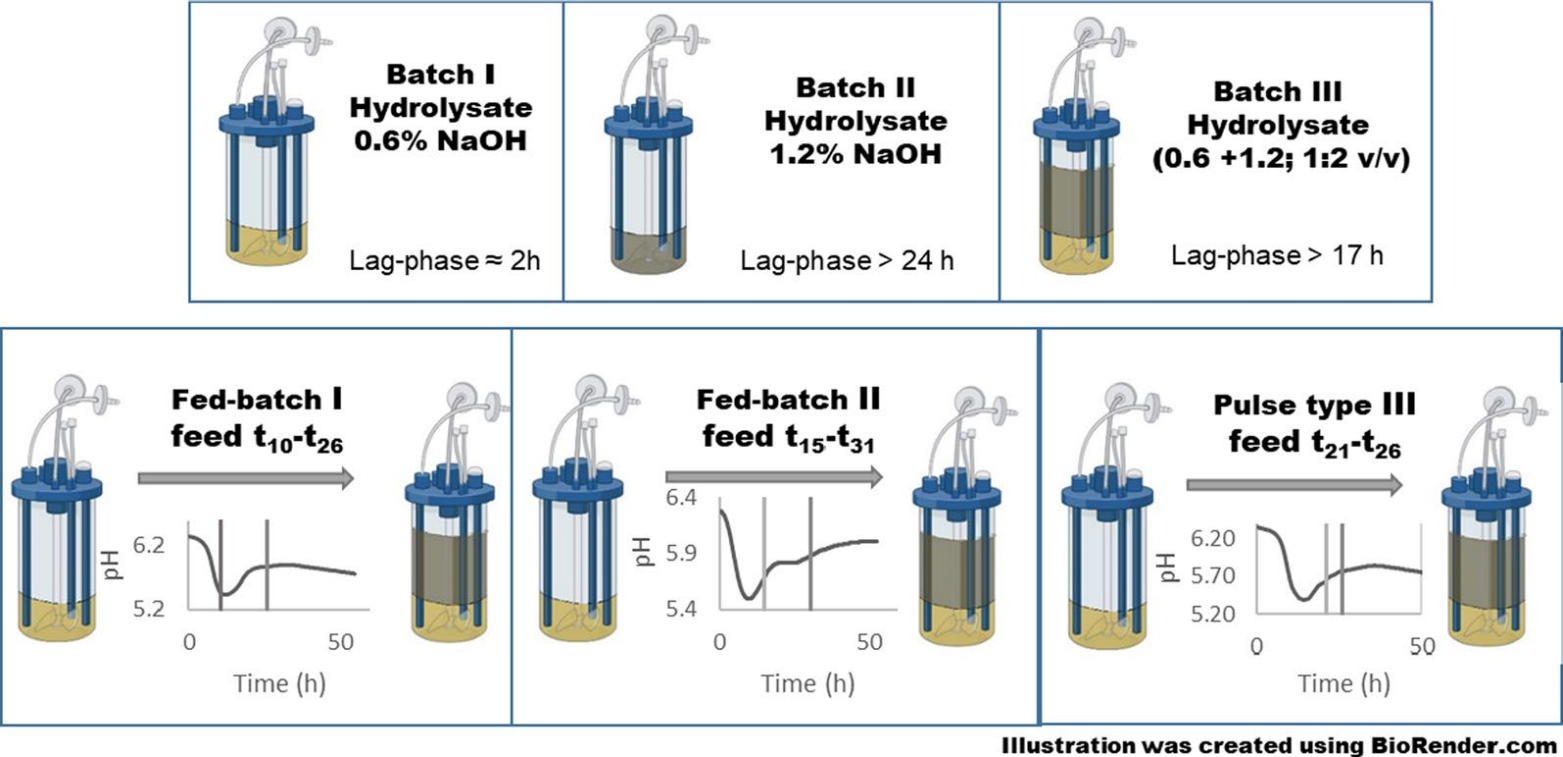

**Supplementary Information:**

The online version contains supplementary material available at 10.1186/s13068-024-02520-6.

## Background

Lignocellulose biomass represents one of the most abundant and promising renewable materials on the planet and, as such, it could serve for sustainable production of bulk chemical commodities via fermentation [1], including solvents such as acetone–butanol–ethanol (ABE) produced by solventogenic clostridia [2–4]. To reap the indisputable environmental benefits of utilizing lignocellulose biomass, a number of obstacles need to be overcome, including the recalcitrant nature of the material [5, 6] and the low sugar content of the resulting hydrolysate due to limitations in the solid-to-liquid phase ratio [7, 8]. There are many methods that assist in loosening the structure of tightly interconnected cellulose, hemicellulose and lignin [9]; biological [10], physical, chemical [11] and their various combinations [12]. More severe conditions of lignocellulose hydrolysis may support the achievement of a higher yield of fermentable saccharides but at the same time, produce a range of inhibitory compounds that negatively influence microbial producers and, subsequently, process performance. Based on the origin and composition of lignocellulose, and the treatment method, the range of inhibitors can be quite extensive [13] and must be considered in the design of an appropriate biotechnological scheme. One of the biggest advantages of employing Clostridium cells in fermentative valorization of lignocellulose lies in their wide detoxification potential [14], which has been demonstrated as an ability to naturally transform a number of inhibitors [15, 16].

Solventogenic clostridia are also able to effectively utilize pentose sugars [17, 18] as well as hexoses [19, 20]. Nevertheless, even this group of microorganisms is only able to tolerate inhibitors to a limited extent and under particular circumstances, and their increased exposure leads to a deterioration in production characteristics [21], including cases when fermentation is inhibited completely [22, 23].

In brief, ABE fermentation consists of two major phases: acidogenic, when predominantly acids (butyric and acetic) are formed and the pH of the fermentation medium decreases, and solventogenic, where, at some threshold point, a metabolic switch can be observed, accompanied by the onset of solvent production and partial acid reutilization. In addition, different phenotypic manifestations are typical for each phase [24]. Distortion or full abortion of this scheme due to the presence of inhibitors results in poor solvent production [25–27], over-acidification of the fermentation medium and cessation of growth.

Among the most common inhibitors released from lignocellulose are furfural and hydroxymethylfurfural, generated from cellulose and hemicellulose [28], phenolic compounds from lignin, acetic acid released by the hydrolysis of hemicellulose acetyl groups and, to a smaller extent, many others. Last but not least, pH adjustment required for particular steps and consequent neutralization increases overall salinity that contributes to the toxicity of lignocellulosic hydrolysates. A partial solution is often found in efficient prevention of inhibitory effects by removal of inhibitors, commonly by discarding of the first liquid fraction after pretreatment followed by additional washing of the solid fraction [29–31], and subsequent hydrolysate detoxification [32]. This leads to a partial loss of soluble carbohydrates from hemicelluloses, an increase in required process steps and increased water and chemical consumption. Another promising approach is to search for or construct more tolerant strains [33–35]; the use of multispecies consortia [36] and strains having inherent enhanced detoxification capacity [14].

A plethora of works were published dealing with ABE production from lignocellulosic materials. Transcriptomic studies, the outputs of which are summarized in our recent review [37], show that responses to inhibitors are rather complex, involving the differential expression of a significant number of genes [25, 38] and considerable changes in metabolism [39]. The more prominent effect was observed, surprisingly, in connection to solvent production rather than to cell growth, while the exact mechanism affecting the regulation of solventogenesis has not been revealed. There are also publications showing that the effect of some inhibitors may not be unequivocally negative. Stimulatory effect of furfural and hydroxymethylfurfural (HMF) on the growth or ABE production was observed when their concentration in the medium was between 0.5–2.0 g/L for various strains of solventogenic Clostridia [40, 41]. Co-presence of organic acids in the fermentation media enhanced significantly the production of total solvents compared to fermentation of medium with phenolic inhibitors but without acids revealing that the synergy of multiple inhibitors can have a surprisingly milder effect [42]. Finally, the action of these substances could vary depending on when they are introduced during ABE fermentation. Pulse addition of furfural and HMF to an exponentially growing culture of *C. acetobutylicum* was more lethal than their addition at the fermentation outset [43]. When *C. beijerinckii* was subjected to furfural stress during the acidogenic growth phase, ABE production was enhanced compared to the control group without stress. However, fermentation ceased when the same strain was exposed to furfural during the solventogenic growth phase [38].

Despite indications that inhibitors may be stimulatory in some cases, fermentative processing of truly crude hydrolysates results in very low solvent production [44, 45] in the vast majority of cases. Thus, interventions are necessary, either in the composition of the fermentation medium in the form of detoxification, masking of the negative effect by the addition of other substances, or intervention in the genome of the production strain. We show here a different approach using the natural detoxification capacity of the production strain.

With this study, we would like to demonstrate that solventogenic clostridia can exhibit varying sensitivities to inhibitors at different stages of growth. Proper timing and a chosen strategy of gradual dosing of the otherwise toxic substrate can significantly mitigate the negative impact of inhibitors and enable its utilization. An optimized fermentation design was introduced for *Clostridium beijerinckii* NRRL-B598 grown on alkali pretreated, and subsequently enzymatically hydrolysed, wheat straw. All input streams were processed as a whole, with no washing steps or detoxification. Finally, the alleviating effect of sequential feeding was verified using a standard culture medium supplemented with the artificial addition of inhibitors. This study is the first to demonstrate the power of the fed-batch strategy for processing crude lignocellulosic hydrolysates prepared without sterilization, detoxification or the need for external carbohydrate addition at any step.

## Materials and methods

### Microorganisms and cultivation conditions

The microorganism used was the bacterial strain *Clostridium beijerinckii* NRRL B-598 [46]. It was stored at a temperature of 4 °C in the form of spore stocks in distilled water. The inoculum was prepared as described in [47] by pipetting 500 µl of heat shocked (80 °C, 2 min) spore suspension in 200 ml TYA medium (20 g/L glucose, 2 g/L yeast extract, 6 g/L tryptone, 0.5 g/L KH_2_PO_4_, 3 g/L ammonium acetate, 0.3 g/L MgSO_4_.7H_2_O and 0.01 g/L FeSO_4_, pH 6.8).

*Impact of individual inhibitors.* Testing of inhibitors was carried out in TYA medium containing a glucose concentration of 45 g/L. Three concentrations of each inhibitory compound: 0.2; 0.4 and 0.6 g/L ferulic or coumaric acids (Sigma-Aldrich) were added prior to inoculation. The experiment was carried out in Erlenmeyer flasks inoculated with a stock spore suspension by pipetting 100 µl of spore suspension into 50 ml of medium supplemented with inhibitor.

*Impact of inhibitor mixture at different growth stages.* To reveal growth stage-dependent susceptibility of Clostridium cells, 20 empty Erlenmeyer flasks residing in the anaerobic chamber were filled with 45 ml of fresh TYA medium containing 40 g/L glucose and inoculated with 5 ml of exponential Clostridium culture. Ethanol (control experiment) or a mixture of coumaric and ferulic acid (dissolved in ethanol) were pipetted into these flasks (five replicates each) at times zero, 7 h, and 23 h (to a final concentration of 0.2 g/L each inhibitor). After a sample t0 was taken, the cotton plugs that sealed the flasks were wrapped with parafilm and cells were cultivated for 96 h.

*Impact of increased salinity at different growth stages.* The inhibitory impact of salt was tested in TYA medium in microtiter plates, which were inoculated with various types of inocula that varied in age, so that cells were in different growth, metabolic or sporulation phases. The salt solution was added to reach a given concentration of Na^+^ ions by pipetting known amounts of Na_2_HPO_4_ water solution (the pH was adjusted to 6.3 with HCl solution) into TYA medium. The inoculum was added in a volumetric ratio of 1:10. Microtiter plates were cultivated in an anaerobic chamber, statically at 37 °C for 24 h, and growth was evaluated based on visual control of colour change using bromocresol purple as a pH indicator.

*Wheat straw hydrolysate preparation.* Wheat straw was collected from the field located in the Czech Republic, after a regular mechanical wheat harvest, and was kept indoors at laboratory temperature until processing. It was first subjected to alkaline and then enzymatic hydrolysis. Before hydrolysis, the straw was milled for 2 min at 4 000 rpm in a knife mill (Grindomix Retsch). The required sample size was mixed with a solution of 0.6% or 1.2% NaOH in a ratio of 1w:10v and hydrolysed in Erlenmeyer flasks on an orbital shaker for 20 h at 80 °C and 150 rpm. The pH was then adjusted to 5.0 ± 0.4 with H_3_PO_4_ and a cellulolytic enzyme complex Cellic CTec2 (Novozymes, Denmark) was added to the mixture (1 ml of enzyme/10 g of straw, corresponding to 35 FPU/g [7]). The mixture was further hydrolysed for 24 h at 50 °C at 150 rpm. The hydrolysate was transferred to 1-L cuvettes and centrifuged (10 min, 6 000 rpm, Sorvall centrifuge). The supernatant was used as the carbon source in all experiments. Hydrolysates were freshly prepared prior to each cultivation and were not stored for longer than 48 h. All steps after alkaline hydrolysis were carried out using sterile equipment and environment, while added chemicals and solutions were not sterilized, neither by heat nor filtration, except for 5 × TYA without glucose (and where indicated also without ammonium acetate). It was prepared as a concentrated solution and added to wheat hydrolysate prior to cultivation, as indicated in Table 1.

**Table 1:** Overview of parameters of batch and fed-batch experiments carried out in bioreactors

Cultivation		Supplement	Inoculum	Final volume	TYA components**
Batch cultivation_Type I					
260 ml of hydrolysate prepared using 0.6% NaOH		45 ml TYA without glucose and ammonium acetate (5x)	70 ml	375 ml	60%
Batch cultivation_Type II					
260 ml of hydrolysate prepared using 1.2% NaOH		45 ml TYA without glucose and ammonium acetate (5x)	70 ml	375 ml	60%
Batch cultivation_Type III					
260 ml of hydrolysate prepared using 0.6% NaOH mixed with 500 ml of hydrolysate prepared using 1.2% NaOH		100 ml TYA without glucose and ammonium acetate (5x)	70 ml	930 ml	54%
Fed-batch_Type I					
Batch phase	260 ml of hydrolysate prepared using 0.6% NaOH	45 ml TYA without glucose (5x)	70 ml	-	-
Feed phase t10.5 – t26	438* ± 10 ml hydrolysate prepared using 1.2% NaOH	37 ml TYA without glucose (5x)	-	850 ml	48%
Delayed Fed-batch_Type II					
Batch phase	260 ml of hydrolysate prepared using 0.6% NaOH	45 ml TYA without glucose (5x)	70 ml	-	-
Feed phase t15-t31	483* ± 10 ml hydrolysate prepared using 1.2% NaOH	43 ml TYA without glucose (5x)	-	901 ml	49%
Pulse addition_Type III					
Batch phase	260 ml of hydrolysate prepared using 0.6% NaOH	45 ml TYA without glucose (5x)	70 ml	-	-
Pulse phase t21-t26	451* ± 10 ml hydrolysate prepared using 1.2% NaOH	41 ml TYA without glucose (5x)	-	867 ml	50%
Fed-batch_Type I—verification experiment with TYA medium					
Batch phase	300 ml TYA without glucose	33 ml sugar solution (250 g/L glucose + 150 g/L xylose)	70 ml	-	-
Feed phase t5.5 – t21.5	441* ± 10 ml modified TYA with inhibitors	–-	-	844 ml	100%

^*^exact volume fed into the bioreactor was counted after determining the residual liquid in the bottle when fed-batch mode was stopped

^**^Amount of TYA medium components without glucose (composition is described above) added concerning final volume

*Batch and fed-batch experiments in bioreactors.* Cultivations were carried out in 1-L Infors laboratory bioreactors. The medium was added to sterile bioreactors according to Table 1 and contents of all reactors, as well as bottles with feed, were bubbled with nitrogen for 10 min prior to the start of the experiments. The pH was adjusted prior to inoculation, to a value of 6.3, and was not controlled during cultivation. The pH of feed was adjusted to 6.0. Each experiment was first performed once and all parameters were monitored during the entire ABE fermentation. Subsequently, each tested mode was repeated in triplicate and only the final outputs were analysed.

**Fig. 1:**
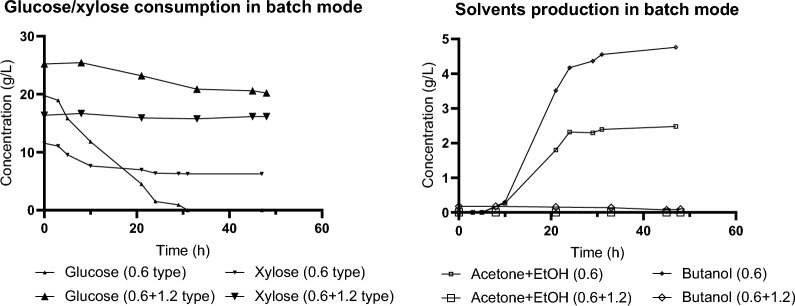
Concentration of glucose, xylose, and ABE during batch cultivation carried out with two types of hydrolysates prepared with different doses of NaOH (0.6 and 1.2%). The graphs show the concentrations of carbohydrates and solvents. Numbers 0.6 and 1.2 in the legend specify the type of hydrolysate used

The verification experiments with TYA medium and artificial addition of inhibitors were carried out to mimic experiments with hydrolysates with no or negligible changes. Only, instead of hydrolysates, TYA medium was prepared without saccharides, these were dosed into a bioreactor from concentrated solution (250 g/L glucose + 150 g/L xylose) prior to inoculation. Inhibitors were resuspended in ethanol and added into bioreactor prior inoculation in case of batch experiment. In case of fed batch, inhibitors were added to the bottles with feed. For saccharides and inhibitors concentration see Table 2 and corresponding charts in Figs. 1, 3, 4, 5, 6.

**Table 2:** Saccharides and inhibitors content in feed streams (in g/L)

	**Glucose**	**Xylose**	**Ferulic acid**	**Coumaric acid**
Hydrolysate 1.2 (Figs. 3–5)	28.2 ± 0.9	16.1 ± 1.9	0.226 ± 0.005	0.208 ± 0.001
TYA feed (Fig. 6)	23.5 ± 0.9	15.0 ± 0.6	0.336 ± 0.011	0.379 ± 0.014

### Analysis of the fermentation medium

Samples were collected during cultivation, frozen immediately, and kept at – 18 °C until analysis. Prior to analysis, samples were thawed, centrifuged (5 min, 6 000 rpm) and supernatants were thermostatically conditioned (24 °C) for conductivity measurements, which were carried out using a Jenway 3540 laboratory conductometer. Samples for HPLC analysis were filtered through a cellulose microfilter with a porosity of 0.2 µm and analysed first for metabolite content on an Agilent Technologies 1200 HPLC and subsequently for inhibitors on an Agilent Series 1260 Infinity UHPLC. To analyse metabolites and sugar content, the following conditions were used: Watrex Polymer IEX H form column, 250 × 8 mm, 8 µm; mobile phase 5 mM H_2_SO_4_, 1 ml/min, injection volume 20 µL, column temperature 60 °C, refractometric detection. Inhibitors were quantified using a Zorbax Eclipse Plus Phenyl-hexyl column, 4.6 × 100 mm, 1.8 Micron, gradient elution with 0.025% H_3_PO_4_ in demi H_2_O (A), acetonitrile (B) flow rate 1.0 ml/min. Temperature was ambient, injection volume 5 µL, and detection was carried out at 320 nm using a DAD detector.

Values for product yields and substrate consumption were calculated from absolute amounts of particular substances according to the following formulas:

$$\text{Substrate consumed }\left(\text{\%}\right) x=(1-\frac{{V}_{Fin} \times {c}_{Fin} }{\left({V}_{t0} \times {c}_{t0}+{V}_{Feed} \times {c}_{Feed}\right)})\times 100.$$

$$\text{Yields }{Y}_{P/S}=\frac{{V}_{Fin }\times {c}_{P\_Fin}}{\left({V}_{t0 }\times {c}_{gl{c}_{t0}}+ {V}_{Feed}\times {c}_{gl{c}_{Feed}}+{V}_{t0 }\times {c}_{xy{l}_{t0}}+ {V}_{Feed}\times {c}_{Xy{l}_{Feed}}\right)-{V}_{Fin}\times ({c}_{gl{c}_{Fin}}+{c}_{xy{l}_{Fin}})},$$

where *t0* stands for time zero and batch mode initial conditions, *Feed* indicates medium fed into bioreactor, *V* is volume and *c* concentration, *Fin* indicates final values, while P means product, S—substrate, glc—glucose and xyl—xylose.

## Results

### Utilization of wheat straw hydrolysates in batch mode

Two different types of alkali pretreated and enzymatically digested hydrolysates from wheat straw were used as a sole carbon source in ABE fermentation carried out under various cultivation modes using *Clostridium beijerinckii* NRRL B-598 as a producer strain. The hydrolysates were used in their crude form. First, the batch mode was tested for hydrolysates prepared with fixed solid loadings of wheat straw (10% w/v), and two different hydroxide concentrations used in pretreatment (0.6% and 1.2%). *C. beijerinckii* was able to growth on hydrolysate prepared with the lower hydroxide concentration (0.6%) showing a negligible lag-phase and standard two-phase metabolism. The final concentrations of solvents were 4.8 g/L of butanol and 6.2 g/L ABE. Double the hydroxide concentration in hydrolysate pretreatment totally inhibited ABE fermentation in batch mode (data not shown as no growth was recorded). Subsequently, both hydrolysate types were mixed in a ratio of approximately 1:2 low:high alkali (for details see Table 1) and subjected to the same experimental conditions. Results of the experiments are shown in Fig. 1. Mixing the hydrolysates did not improve fermentation performance and no butanol production was recorded*.*

### Inhibitory compounds in wheat straw hydrolysate—phenolic acids and salinity

To determine what compounds inhibited clostridial growth, both types of hydrolysates were analysed for the presence of furan derivatives and phenolic substances typical for lignocellulose treatment. Neither furfural nor hydroxymethylfurfural were detected. Among the phenolic substances, the most abundant were coumaric and ferulic acids. Their concentrations in the hydrolysate prepared with a lower hydroxide concentration were 0.157 ± 0.004 g/L and 0.194 ± 0.007 g/L, respectively. Wheat straw hydrolysate prepared with 1.2% NaOH contained 0.226 ± 0.005 g/L of coumaric acid and 0.208 ± 0.001 g/L ferulic acid.

The impacts of these two inhibitors on glucose consumption, acid and butanol production were tested in small-scale batch experiments, where inhibitors were added to TYA medium prior to inoculation. The results are summarized in Table 3. The inhibitory effect of both substances was evident from the lowest concentration tested. This concentration corresponded to the levels detected in wheat straw hydrolysates. Increasing concentrations of inhibitors led to decreased butanol formation, and complete inhibition occurred when 0.6 g/L of inhibitor were added. This observation proves the inhibitory effect of ferulic and coumaric acid but does not provide an explanation for the inability of *C. beijerinckii* to grow on hydrolysate with concentrations of these compounds around 0.4 g/L (as the sum of both).

**Table 3:** The effect of coumaric or ferulic acids on ABE fermentation. Values in the table show mean values and standard deviations of three biological replicates for glucose consumption and the amount of butyric acid and butanol formed after 70 h of static fermentation with *C. beijerinckii NRRL B598*

	Glucose consumption (% from 45 g/L)	Butyric acid (g/L)	Butanol (g/L)
0.2 g/L Coumaric ac	55 ± 1	0.6 ± 0.1	5.0 ± 0.1
0.4 g/L Coumaric ac	27 ± 9	1.4 ± 0.2	1.4 ± 0.5
0.6 g/L Coumaric ac	0	0.0	0.0
0.2 g/L Ferulic ac	44 ± 1	0.8 ± 0.0	3.6 ± 0.1
0.4 g/L Ferulic ac	36 ± 2	1.4 ± 0.1	2.7 ± 0.3
0.6 g/L Ferulic ac	0	0.0	0.0
Control	57 ± 2	0.7 ± 0.0	5.4 ± 0.2

Additional or synergistic effects of increased salinity were considered and explored in a subsequent experiment. Inhibitory activity was tested using the microdilution method and only growth ability was evaluated. While NaOH and H_3_PO_4_ were used to adjust the pH in all steps of hydrolysate preparation, Na_2_HPO_4_ was used in screening for the inhibitory effect. To reflect the possible variation in sensitivity of different culture stages, sensitivity to salts was tested for four different types of inoculum, varying in age: *early acidogenic* (very early growth stage—shortly after growth was visually detected), *late acidogenic* (culture 6 h older than the previous one), *mid-solventogenic* (culture from the second day in which a decrease in gas production was already visually observed), *late solventogenic* (the second day culture 6 h older than the previous one). The Na^+^ concentration tested was up to 0.34 M. As expected, the effect of salts was growth stage dependent, with the early acidogenic phase being the most sensitive to increased salinity, see Table 4. Late acidogenic to mid-solventogenic stages seem to be the least susceptible to the inhibitory effect of salts. This experiment also confirmed that increased salinity may contribute to the inhibitory effect.

**Table 4:** Inhibitory impact of increased salinity on growth of *C. beijerinckii* NRRL B-598 at various stages of their life cycle

Concentration of Na (mol)/ Inoculum age*	0.00	0.21	0.25	0.28	0.31	0.34
*Early acidogenic (18 h)*	+	-	-	-	-	-
*Late acidogenic (24 h)*	+	+	+	+	+	+
*Mid solventogenic (42 h)*	+	+	+	+	+	±
*Late solventogenic (48 h)*	+	+	+	+	±	-

For each salt concentration and inoculum age, 8 wells were tested:

(-) no growth was observed in all 8 parallels with identical conditions

( +) growth was observed in all 8 parallels with identical conditions

( ±) both patterns or insignificant growth were observed within 8 parallels

^*^inoculum age indicates the time difference between the inoculation of spore suspension into TYA medium and the time when an already growing culture was transferred into wells with various concentrations of salts. Particular growth stages refer to the status of the culture transferred into individual wells

### Testing the inhibitory effect of phenolic compounds at different stages of culture growth

**Fig. 2:**
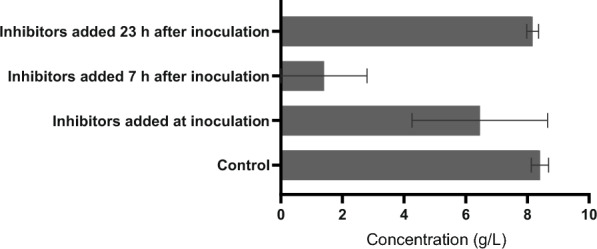
Comparison of the total solvent concentration produced (acetone plus butanol) for different inhibitor addition times. The columns correspond to the mean values and standard deviations are depicted as error bars for the five biological replicates

The previous results indicate that the sensitivity of clostridia might be dependent on the particular stage of growth. To verify this hypothesis, inhibitors were added to the Erlenmeyer flasks at various stages of growth and production. To minimize the impact of culture variability, all experiments were started from a single inoculum and cultivated under exactly the same conditions. The concentration of coumaric and ferulic acids was constant at 0.2 g/L each (added together, so that final concentration of inhibitors was 0.4 g/L). Because the inhibitors were dissolved in ethanol, the total solvent production was not evaluated as the sum of ABE, but only for butanol and acetone (AB). The resulting values are depicted in Fig. 2, together with an indication of the variability of the results obtained in five identical parallels. This graph clearly demonstrates that there are physiological stages of *C. beijerinckii* culture that are almost unaffected by the addition of inhibitors, those that are very sensitive to the addition of inhibitors, and finally those that show extreme variability in relation to inhibitors.

The concentrations of glucose and metabolites at the time of addition of inhibitors and the concentrations at the end of the experiment are summarized in Table 5. From the results, it appears that the physiological stages of *C. beijerinckii* cells show the highest resistance to antimicrobial substances from late acidogenesis to early solventogenesis. Therefore, this hypothesis was subsequently used to design a technological process that would allow for the fermentation of toxic hydrolysates.

**Table 5:** Concentration of glucose and metabolites at the moment of addition of inhibitors and at the end of the cultivation (96 h)

in g/L		Control	Inhibitors added at inoculation	Inhibitors added 7 h after inoculation	Inhibitors added 23 h after inoculation
Glucose	Start	37.1 ± 0.6	37.1 ± 0.5	35.4 ± 0.7	29.2 ± 1.0
	End	8.8 ± 0.9	18.6 ± 6.1	31.8 ± 0.6	10.6 ± 0.8
Acetic acid	Start	2.5 ± 0.3	2.3 ± 0.1	2.5 ± 0.1	1.8 ± 0.1
	End	1.2 ± 0.3	1.7 ± 0.1	2.5 ± 0.1	1.1 ± 0.0
Butyric acid	Start	0.0 ± 0.0	0.0 ± 0.0	0.7 ± 0.1	0.4 ± 0.1
	End	1.2 ± 0.4	1.2 ± 0.4	2.1 ± 0.1	0.7 ± 0.1
Ethanol*	Start	1.4 ± 0.1	1.0 ± 0.2	1.2 ± 0.1	1.1 ± 0.1
	End	1.3 ± 0.0	1.0 ± 0.0	1.0 ± 0.0	1.1 ± 0.1
Acetone	Start	0.0 ± 0.0	0.0 ± 0.0	0.0 ± 0.0	0.9 ± 0.1
	End	1.7 ± 0.1	1.4 ± 0.6	0.4 ± 0.0	2.0 ± 0.1
Butanol	Start	0.0 ± 0.0	0.0 ± 0.0	0.0 ± 0.0	1.7 ± 0.1
	End	6.7 ± 0.2	5.1 ± 1.6	1.0 ± 0.2	6.2 ± 0.1

^*^The increased ethanol concentration at the beginning of the experiment is due to the addition of inhibitors that were dissolved in ethanol, since DMSO commonly used for these purposes interferes with the HPLC determination of butyric acid and acetone under the chosen separation conditions

Coumaric and ferulic acids (each at 0.2 g/L, dissolved in ethanol) were added to the fermentation medium at various stages of growth. The numbers in the table correspond to the mean value and standard deviation of the five biological replicates carried out in TYA medium with 40 g/L initial concentration of glucose

### Testing different feeding strategies

A fed-batch cultivation was suggested as a possible approach to cope with the toxicity of inhibitory compounds in crude hydrolysates. In the following experiments three different strategies were tested. All cultures started in batch mode with hydrolysate prepared using 0.6% NaOH and differed in the timing of dosing of toxic hydrolysate (prepared using 1.2% NaOH). The timing was chosen according to the on-line monitored pH values. In the first experiment, the feed was started just before the pH began to rise due to the metabolic switch (*Fed Batch_Type I*). In the second scheme, the feed was started only after a demonstrable rise in pH indicating active acid reutilization (*Delayed Fed Batch_Type II*). In both cases, the dosing of the toxic hydrolysate was gradual and lasted 16 h. In the third scheme, the later start of feeding was chosen, but toxic hydrolysate was pumped at a 3 times higher speed (*Pulse addition_Type III*). The resulting ratio of hydrolysates at the end of the fed-batch phase was the same for all experiments, i.e. 1:2 (details are summarized in Table 1).

**Fig. 3:**
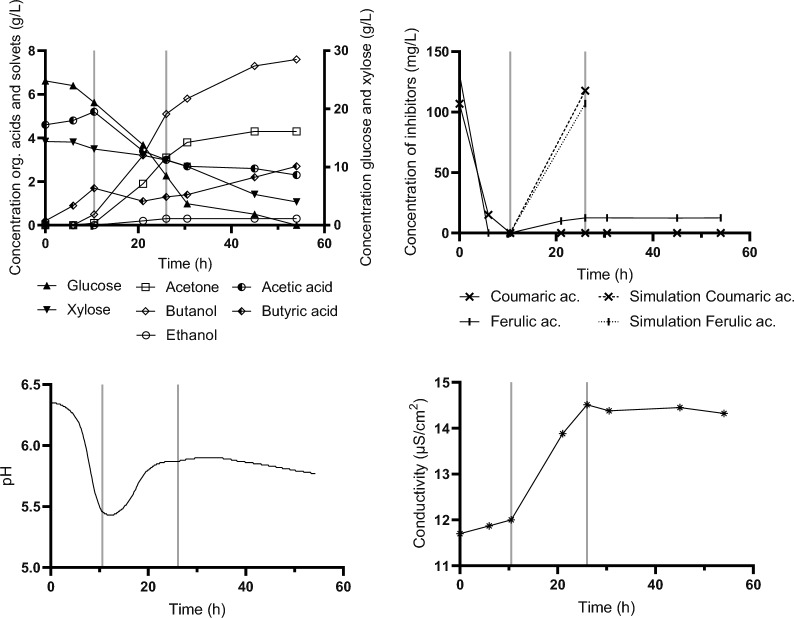
Concentration of glucose, xylose and metabolites, in medium during fed-batch cultivation (*Fed-batch_Type I*)—upper left. Upper right—concentration of inhibitors in medium, dashed lines indicate the theoretical concentration of inhibitors in the bioreactor based on flow rate of feed (if no degradation occurs). The lower part shows pH recorded on-line (left) and conductivity (right). Black vertical lines indicate the start and end of feeding. Interim data are presented for one bioreactor. Summary data for three replicates with standard deviations indicated are shown in Table 6

The first scheme (*Fed Batch_Type I*), started when a change in pH indicated an approaching switch to solventogenesis (apparent reduction in rate of pH decrease). This was observed at the 10.5th hour of batch cultivation (Fig. 3 lower-left). Feeding rate was set to approximately 30 mL/h, was stopped at the 26th hour, and cultivation was continued in batch mode until the apparent cessation of microbial activity. All glucose and 76.4% of xylose were utilized and the final concentration of butanol achieved was 7.6 g/L (12.1 g/L ABE). Both glucose and xylose were consumed simultaneously, although at a higher rate for glucose. The time courses of changes in sugar and metabolite levels are shown in Fig. 3. Both phenolic inhibitors present in the hydrolysate were degraded within the early growth stage and efficient degradation was also observed during the feeding period. The upper right chart in Fig. 3 shows ferulic and coumaric acid concentrations during the experiment while the dotted line simulates an increase in their concentrations when they were not metabolized. Finally, the lower right chart in Fig. 3 shows a gradual increase in medium salinity allowing cells to adapt. This fed-batch design allowed successful fermentation in medium that originally did not enable growth of *C. beijerinckii* at all.

**Fig. 4:**
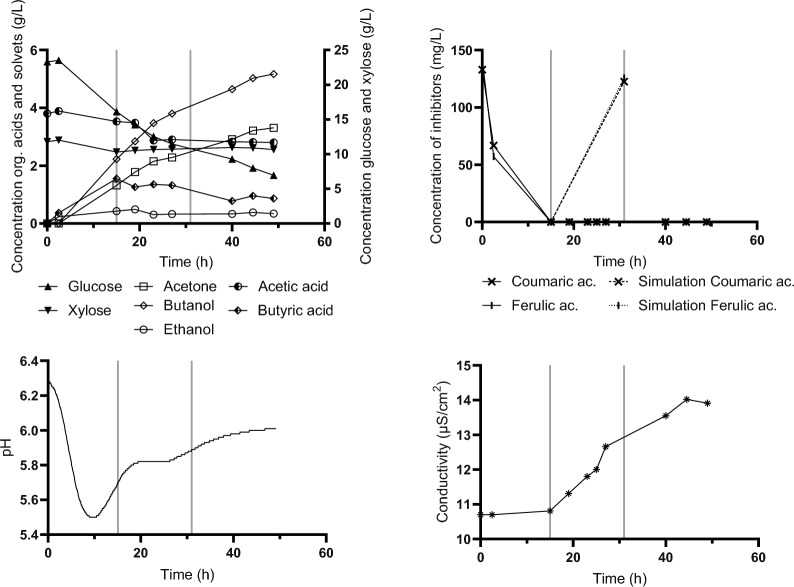
Concentration of glucose, xylose and metabolites in medium during fed-batch cultivation (*Delayed Fed-batch_Type II*)—upper left. Upper right—concentration of inhibitors in medium, dashed lines indicate the theoretical concentration of inhibitors in the bioreactor based on flow rate of feed (if no degradation occurs). The lower part shows pH recorded on-line (left) and conductivity (right). Black vertical lines indicate the start and end of feeding

The second scheme tested (*Delayed Fed Batch_Type II*) copied the first one while the start of feeding was initiated a few hours later (in 15th hour), when the pH curve clearly showed extensive acid reutilization (see Fig. 4—lower left) and hence putative solventogenesis. The other parameters were kept similar to the first strategy. Results are shown in Fig. 4. 76.7% of available glucose and 22.7% of xylose were utilized, resulting in a final butanol concentration of 5.3 g/L (9.1 g/L ABE).

**Fig. 5:**
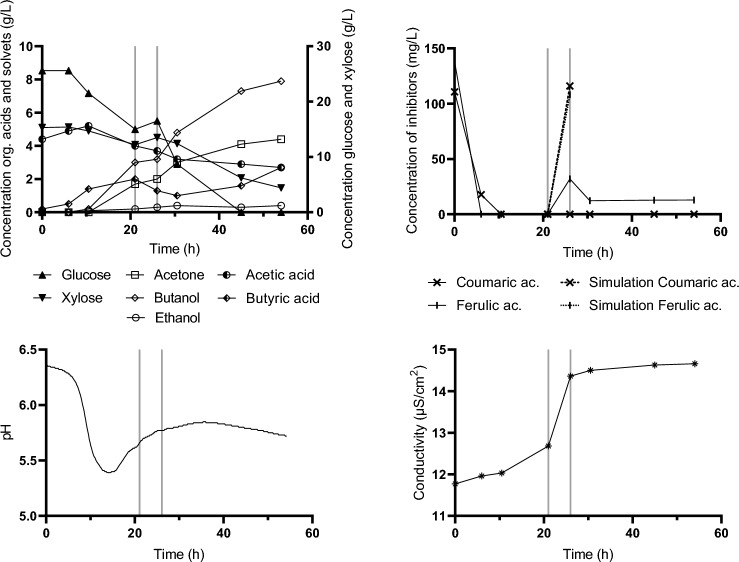
Concentration of glucose, xylose and metabolites in medium during pulse type cultivation (*Pulse addition_Type III*)—upper left. Upper right—concentration of inhibitors in medium, dashed lines indicate theoretical concentrations of inhibitors in the bioreactor based on flow rate of feed (if no degradation occurs). The lower part shows pH recorded on-line (left) and conductivity (right). Black vertical lines indicate the start and end of feeding

In the third scheme (*Pulse addition_Type III*), a later start of feeding was chosen (at 20th hour) and toxic hydrolysate was fed at approximately a 3 times higher speed resembling a pulse feed rather than a fed-batch. Data are shown in Fig. 5. The overall time of cultivation was similar to previous experiments while results were very promising with the highest butanol concentration reached (7.9 g/L; ABE 12.8 g/L).

To verify the robustness and repeatability of the suggested concepts, all experimental strategies were repeated in three parallel bioreactors under similar conditions. The resulting values, showing the mean and standard deviations, are summarized in Table 6. Similar to the inhibitor testing (Fig. 2), we observed a substantial variability in results for multiple repetitions of the selected feeding strategies, with stable and high performance achieved only when the toxic hydrolysate feeding was started close to the metabolic switch (*Fed-batch_Type I*).

**Table 6:** Yield of butanol and total ABE production, glucose, xylose and acetic acid consumption in fed batch and pulse cultivations

	Fed-batch_Type I	Delayed Fed-batch_Type II	Pulse addition_Type III
Glucose consumption (%)	100.0 ± 0.0	65.5 ± 8.0	87.3 ± 14.4
Xylose consumption (%)	88.3 ± 4.5	21.6 ± 4.4	37.1 ± 20.1
Acetic acid consumption (%)	36.8 ± 1.8	61.5 ± 6.9	10.1 ± 37.5
Butanol (g/L)	7.0 ± 0.0	4.1 ± 0.8	4.3 ± 1.7
ABE (g/L)	12.2 ± 0.1	7.2 ± 1.4	7.0 ± 3.2
Y ABE (g/g)	0.31 ± 0.00	0.34 ± 0.02	0.30 ± 0.04
Y Butanol (g/g)	0.18 ± 0.00	0.19 ± 0.02	0.18 ± 0.02

Even though non-detoxified crude wheat straw hydrolysate was used, results were comparable with those reached using laboratory medium, pure glucose and strain *C. beijerinckii* NRRL B-598 (see e.g. [36, 37] and/or Supplementary material_Fig. S1 for glucose and xylose mixture). Moreover, the butanol concentration was 46% higher than in the batch experiment carried out with the hydrolyzate prepared with 0.6% NaOH (Fig. 1). On the other hand, the other two feeding regimes failed to provide stable repeatable results. Even though the yield coefficients were little affected, final titers varied considerably within three parallel bioreactors. One of our hypotheses for decreased solvent production is that the addition of a more basic hydrolysate (pH 6.0) after the metabolic switch may have reduced the need to adjust the pH by the acids reutilization, consequently, more acids could have been produced at the expense of solvents.

In addition to metabolites, presence of inhibitors was analysed for all experimental settings. It was observed that in any experiment where clostridial growth was recorded, the concentration of inhibitors dropped to zero or near-zero values, independent of the resulting solvent production. This verified the robust capability of the strain to transform coumaric and ferulic acid initially present or added to the medium.

### Verification of fed batch concept for eliminating inhibitory effect of coumaric and ferulic acid

**Fig. 6:**
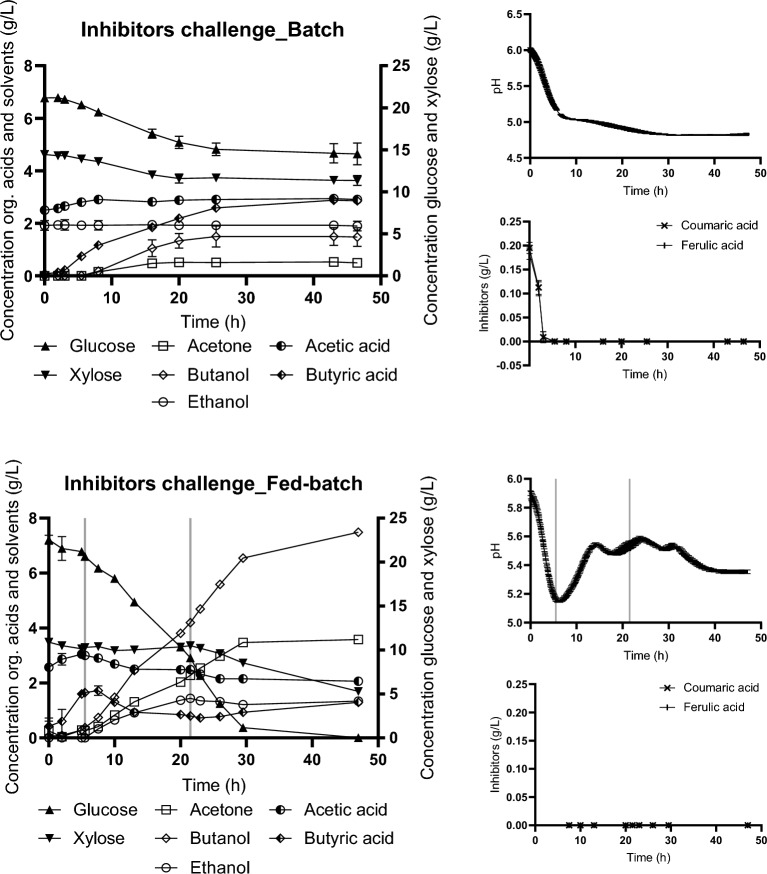
Concentration of glucose, xylose and metabolites in medium during batch (upper part) and fed-batch (lower part) for culture cultivated in modified TYA medium and challenged with ferulic and coumaric acid. The charts on the right show pH recorded online and inhibitor concentrations in samples withdrawn during experiments. Black vertical lines indicate the start and end of feeding. Error bars represent standard deviation of three replicates

Finally, to verify the hypothesis that appropriately chosen feeding strategy can eliminate the negative effect of lignocellulose-derived inhibitors in ABE fermentation, batch and fed-batch cultivations were carried out in modified TYA medium with artificial addition of ferulic and coumaric acid. The experiments mimicked those with crude hydrolysate using the best strategy (*Fed-batch_Type I).* The sugar composition was set to resemble glucose and xylose content in hydrolysates, 25 and 15 g/L resp. Both ferulic and coumaric acids were added together. In case of batch experiments inhibitors were added prior to the inoculation in a concentration of 0.2 g/L each. For fed-batch experiments, cultivation was started as a batch without inhibitors, and ferulic and coumaric acids were added to feed in concentrations corresponding to 0.2 g/L each calculated for the final volume of medium in bioreactor at the end of experiment (Table 2). The course of the experiments is shown in Fig. 6.

In the batch culture experiment, where ferulic and coumaric acid (0.2 + 0.2 g/L) were added prior to inoculation, the final butanol concentration was only 1.5 ± 0.4 g/L. Glucose and xylose utilization was 31 ± 6%, and 21 ± 2%, resp. The implementation of a fed-batch approach yielded significant enhancements. Sequential feeding of medium with the inhibitors (ferulic and coumaric acids corresponding to 0.2 + 0.2 g/L in final bioreactor volume) initiated during metabolic switch resulted in 500% increase in butanol concentration (over batch regime), reaching 7.5 ± 0.1 g/L of butanol. Glucose and xylose utilization were 100% and 65.5 ± 0.6% resp. These numbers reached by fed-batch strategy are fully comparable to experiments without inhibitors at all under similar conditions (TYA medium containing mixture of glucose and xylose, batch cultivation of strain *C. beijerinckii* NRRL-B598 (see Supplementary Fig. 1)).

A comparison of the final butanol concentrations achieved in selected studies before and after intervention leading to a reduction in the negative effect of inhibitors is shown in Table 7. These data clearly show that the use of crude, non-detoxified hydrolysates leads to reduced butanol production and that either the culture medium or the production strain needs to be modified for effective use of lignocellulosic hydrolysates. In this study, we have shown that both can be circumvented by optimized dosing of an otherwise toxic medium.

**Table 7:** Comparison of butanol concentrations obtained with similar studies and using different approaches to deal with the negative effects of inhibitors

Material	Sugar content (g/L):	Inhibitors (g/L):	Butanol prior intervention (g/L)	Method to overcome negative effect of inhibitors	Butanol after intervention (g/L)	Improvement %	Strain	Refs
Wheat straw, H_2_SO_4_ pretreatment, enzyme hydrolysis	Glucose: 20.59 Xylose: 14.11	HMF: 2.52 Furfural: 1.96	1.18	Detoxification: strong acid cation exchange resin	7.42	518%	*C. acetobutylicum* CICC 8012	[44]
Wheat straw, H_2_SO_4_ pretreatment, enzyme hydrolysis	Glucose: 21.05 Xylose: 20.57 Cellobiose: 3.21 Arabinose: 2.28 Galactose: 1.82 Mannose: 1.94	HMF: 2.52 Furfural: 1.96 Syringaldehyde 0.13 Vanillin 0.16 Ferulic acid: 0.37 Coumaric acid: 0.15	5.17	Addition of sodium sulfide	7.63	48%	*C. acetobutylicum* CICC8012	[48]
Non-detoxified hydrolysate of corn fibre treated with dilute sulfuric acid	Glucose: 4.7 Xylose: 44.6 Arabinose: 3.3	TPC 2.77 HMF 0.38 Furfural 0.66	1.6	Strain mutation and selection for increased inhibitors tolerance	6.8	425%	*C. beijerinckii* NCIMB 8052/ *C. beijerinckii* IB4	[49]
Wheat straw, NaOH pretreatment, enzyme hydrolysis	Glucose: 39.75 Xylose: 12.21	NA	NA	Separation and washing of solids after alkali pretreatment	7.05	NA	*C. acetobutylicum* 824	[31]
Rice straw, H_2_SO_4_ pretreatment, enzyme hydrolysis	Glucose: 22.9 Xylose: 15.8	TPC 0.7	0	Electrochemical detoxification	8.0	NR	*C. beijerinckii* NCIMB 8052	[45]
Wheat straw, NaOH pretreatment, enzyme hydrolysis	Glucose batch: 21.4 Xylose batch: 13.2 Glucose feed: 28.2 Xylose feed: 16.1	Ferulic acid batch: 0.16 Coumaric acid batch: 0.19 Ferulic acid feed: 0.23 Coumaric acid feed 0.21	0	Gradual dosing of hydrolysate in fed-batch mode	7.0	NR	*C. beijerinckii* NRRL B598	This study
TYA medium with ferulic and coumaric acid	Glucose batch: 22.6 Xylose batch: 11.1 Glucose feed: 23.5 Xylose feed:15.0	Ferulic acid batch: 0.2 Coumaric acid batch: 0.2 Ferulic acid feed: 0.34 Coumaric acid feed 0.38	1.5	Gradual dosing of inhibitors in fed-batch mode	7.5	500%	*C. beijerinckii* NRRL B598	This study

TPC total soluble phenolic compounds; NA- data not available; NR- not relevant calculation against zero production

## Discussion

Our results indicate that the toxicity of lignocellulose-derived inhibitors can be overcome by the appropriate selection of cultivation conditions and design of a fermentation strategy. Two major assumptions were used in suggesting the proposed technological scheme: firstly—the susceptibility of Clostridium cells to lignocellulose-derived inhibitors would vary alongside their live cycle. Secondly, sequential intermittent feeding can alleviate inhibitor toxicity owing to the clostridia ability to transform them into less toxic compounds.

Solventogenic clostridia belong to microorganisms with high biodegradation potential of lignocellulose-derived inhibitors [14] while the best described is the transformation of furfural and HMF into their respective alcohols using aldo/keto reductase (AKR) and short-chain dehydrogenase/reductase (SDR) [15, 38, 50]. Similarly, AKR integration into the *C. beijerinckii* NCIMB 8052 genome led to increased tolerance to phenolic substances 4-hydroxybezaldehyde, and syringaldehyde [33]. In our study, the abundance of ferulic and coumaric acids was determined in alkali-treated wheat straw hydrolysates. Detoxification of coumaric acid by *C. beijerinckii* NCIMB 8052 was shown to takes place through its reduction into corresponding 3- phenylpropionic acids [16] and a similar reduction to the corresponding propionic acid may be responsible for the biotransformation of ferulic acid as was shown for selected members of *Clostridiales* [51]. All of these reactions are assumed to use intracellular pool of reduced NADH or NADPH cofactors, which in turn leads to their deficiency for the production of solvents.

Rapid depletion of both ferulic and coumaric acid occurred shortly after metabolic activity was manifested and a growing culture was able to transform levels gradually dosed into the bioreactor. Published data suggest an average inhibitory concentration of ferulic and coumaric acids, varying between strains, of approximately 0.5 g/L in the case of a single substance presence in the medium [41, 49, 52, 53]. *C. beijerinckii* strain NCIMB 8052, which is closely related to strain NRRL B-598, showed a very strong inhibition of solvent production by the addition of 0.5 g/L of these compounds, with a resulting butanol concentration of less than 1 g/L [49, 54]. At a coumaric acid concentration of 0.5 g/L, *C. beijerinckii* NCIMB 8052 production was inhibited by 98.8% [45], *C. acetobutylicum* 824 by 90% [40], and *C. beijerinckii* BA101 by 30% [41]. Exposure of *C. beijerinckii* BA101 to 0.3 g/L ferulic acid in a batch fermentation resulted in little or no ABE production [41]. Addition of 0.5 g/L ferulic acid to culture of *C. beijerinckii* NCIMB 8052 resulted in butanol production decrease by 52.1% [45]. These results are in compliance with our findings but does not provide an explanation of total growth inhibition by the wheat straw hydrolysate used. Inhibitors originating from the processing of lignocellulose are variable; it is never just one specific substance but interactions between several, accompanied by additional factors. Elevated salinity levels could potentially hinder growth in our experiments, as solventogenic clostridia tend to be quite susceptible to its effects [55–57]. Together, combinations of inhibitors present in the fermentation mixture might have different effects than a model solution [42]. Gradual dosing of the toxic substrate allows the bacteria to activate expression of required genes and smoothly adapt to the changing environment. If it simultaneously removes certain inhibitors, as was the case for ferulic and coumaric acids, it reduces the overall burden represented by a “deadly” mixture of crude non-detoxified hydrolysate or artificially added inhibitors.

Recent studies unequivocally demonstrate that adopting a "piecemeal" approach generally enhances fermentation performance, whether through intermittent but delayed additions or continuous feeding methods. Chacón et al. [22] used a fed-batch strategy for utilizing a non-fermentable substrate by gradually dosing hydrolysate into the medium based on sugarcane molasses. Molasses was used in the first stage of ABE fermentation while hemicellulose hydrolysate was fed into the fermentation mixture after 24 h. The best results were obtained for a molasses:hydrolysate ratio of 3:1, but the conversion decreased with an increasing ratio of hydrolysate. Adesanya et al. [58] demonstrated improved fermentability of non-detoxified hydrolysate by splitting it into two portions (30:70), where 70% were intermittently fed into a culture already propagated in a smaller volume (30%) of switch grass hydrolysate.

Moreover, the crucial effect of the timing of inhibitor addition on the subsequent culture response has been reported for a furfural challenged culture by Zhang et al. [38]. While its supplementation during acidogenic phase slightly improved ABE production, the similar dose during solventogenesis had a detrimental effect and clear differences were also evident from the transcriptomic profile of differentially expressed genes. The advantage of a fed-batch strategy to decrease the inhibitory effect of substrate might theoretically turn into a disadvantage, especially in the case of ABE fermentation, owing to the accumulation of toxic fermentation products in later stages. This is based on the combined effect of increasing concentrations of inhibitory and toxic products [38], thus Qureshi et al. [23] integrated sequential feeding of toxic hydrolysate into a non-toxic medium with in situ butanol recovery.

Proper timing of fed batch might also benefit from increased capability of cells to efflux inhibitors, so that cells earn more time for their transformation while inhibitors are kept out of the cell interior. Increased efflux pump activity is associated with improved capability to cope with xenobiotics in general. Insertion and overexpression of certain components of efflux pumps from less susceptible strains might considerably enhance tolerance to different inhibitors [59]. Moreover, genes for efflux pumps are commonly upregulated in response to lignocellulose-derived inhibitors and among them those of the ABC type [38, 60]. The highest efflux pump activity during ABE fermentation was detected just prior to the metabolic switch, with a predominant representation of ABC-type pumps [61], which is in agreement with our observation that late acidogenic stages belong to the least susceptible stages to inhibitor impacts.

Unfortunately, the fed-batch itself does not prevent “acid-crash”, a phenomenon in which acid production is prioritized over solvent production [62] and leads to very low final ABE concentrations and premature termination of fermentation. Definitely, the presence of inhibitory substances of different natures promotes a disability to produce solvents at high yields [33, 41, 63–65] and induces an “acid-crash-like” effect. In our study, this could be clearly seen on the example of the artificial addition of a mixture of ferulic and coumaric acid at the beginning of cultivation (Fig. 6). Su et al. [66] successfully integrated intermittent feeding together with prevention of acid crash by temporary pH adjustment. This might provide another explanation of our results reached for fed-batch regime, where the best and stable outputs were reached when toxic hydrolysate feeding was started in late acidogenesis. The medium fed into the bioreactor had been adjusted to pH 6.0, so that it slightly equilibrated the pH in the bioreactor in the critical interval of metabolic switch.

Regarding the efficient utilization of carbon sources present in hydrolysates, the experimental design matters too [67, 68]. The most abundant monosaccharides in wheat straw hydrolysate were glucose and xylose in an approximate ratio of 25:15 (g/L) in this study. Glucose and xylose were utilized to various extents depending on cultivation scheme while xylose was utilized directly from the beginning of the experiment, even though at a lower rate than glucose. It was clearly demonstrated that by selection of appropriate cultivation design, 100% of glucose and up to 88% amount of xylose might be consumed after 54 h of fermentation of undetoxified hydrolysate using a wild-type strain. The xylose consumption was even higher than the median value calculated for lignocellulose hydrolysates in a comparative study compiled by Birgen et al. [69] from multiple studies. The xylose utilization median was 80.8% including the detoxified hydrolysates and the same applies for ABE production with the median of 9.33 g/L [69]. Nevertheless, a comparison of different strains, hydrolysate types and their sources must be performed very cautiously since each strain has different production characteristics and maximum concentrations that it can reach.

## Conclusion

In this study, we have demonstrated that by proper bioprocess design, it was possible to reach fully comparable outputs for toxic substrates as for culture medium without inhibitors. We have used the strain we have the most experience with, *C. beijerinckii* NRRL B-598, integrated various contributions aimed at eliminating the inhibitory effect, and suggested robust and repeatable production of ABE using otherwise toxic substrate. The crude hydrolysate preparation omitted many common, economically and ecologically burdensome interventions, such as the first biomass separation after pre-hydrolysis, washing the solid fraction, sterilization and detoxification. We are convinced that the proposed concept is applicable to a wide range of hydrolysates and production strains. Thus, among others, this study proposes an option for a feasible process of lignocellulose transformation to ABE that complies with the concept of a circular economy.

### Supplementary Information

Supplymentary material

## Data Availability

The data that support the findings of this study are included in this published article and its supplementary information files.
