# Supplementary material for: Application of fed-batch strategy to fully eliminate the negative effect of lignocellulose-derived inhibitors in ABE fermentation
Source: Biotechnol Biofuels Bioprod. 2024 Jun 25;17:87. doi: 10.1186/s13068-024-02520-6 (PMC11197323; doi:10.1186/s13068-024-02520-6)
Supplement: Supplementary file 1 — Supplymentary material [file 13068_2024_2520_MOESM1_ESM.pdf]

Title: Application of fed-batch strategy to fully eliminate the negative effect of lignocellulose-derived inhibitors in ABE fermentation

Barbora Branska*, Kamila Koppova, Marketa Husakova, Petra Patakova

Supplementary Figure 1:

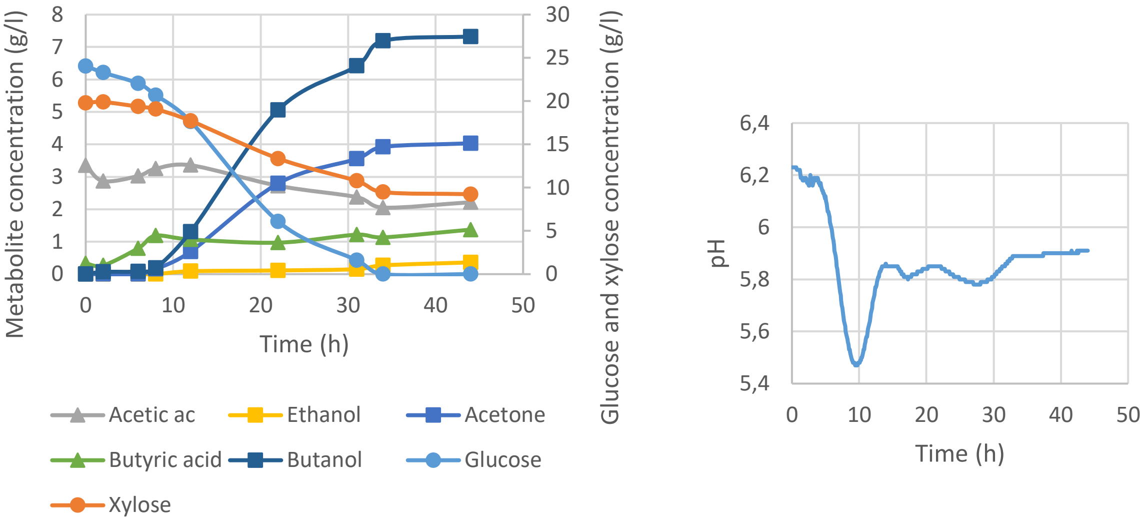

Figure S1: Concentration of glucose, xylose and metabolites (left) and pH changes (right) during batch cultivation using TYA medium and a mixture of glucose and xylose 25:15 in the same volume and bioreactors where experiments with lignocellulose were conducted.
